# Complete genome sequence of the *Listeria innocua* lytic bacteriophage LIS04

**DOI:** 10.1128/mra.00249-25

**Published:** 2025-04-29

**Authors:** Siyeon Park, Yoonjee Chang

**Affiliations:** 1 Department of Food and Nutrition, College of Science and Technology, Kookmin University, Seoul, South Korea; Portland State University, Portland, Oregon, USA

**Keywords:** *Listeria*, *Listeria innocua*, bacteriophage, genomic characterization, biocontrol agent

## Abstract

The *Listeria innocua* American Type Culture Collection 51742-specific phage LIS04, isolated from paddy water, belongs to *Caudoviricetes* class. Phage LIS04 has a 181,606 bp double-stranded DNA with 37.09% guanine-cytosine content and 217 predicted open reading frames. Genomic analysis confirmed its lytic cycle and low similarity to known phages.

## ANNOUNCEMENT

*Listeria innocua* is a gram-positive bacterium commonly found in food-processing environments (1). Although non-pathogenic, its increasing antibiotic resistance highlights the need for control strategies (1). While *Listeria monocytogenes* bacteriophages (phages) are well studied, *L. innocua*-specific phages remain understudied (2–4). We report the isolation and characterization of LIS04, a novel lytic phage specific to *L. innocua*.

LIS04 was isolated from paddy water collected on 11 April 2023 in Seosan, South Korea (36.7834°N, 126.4522°E), using a double-layer assay (5). Single-plaque purification was performed seven times (5). For phage amplification, the host culture was incubated until early exponential phase and cultivated with LIS04 at a multiplicity of infection of 1 at 37°C for 4 h under aeration (6). *L. innocua* ATCC 51742 served as the host for both phage isolation and amplification, cultivated in brain heart infusion broth and agar. Host range was assessed against 20 bacterial strains via spot assay (5), and details are provided in Table 1.

**TABLE 1:** The antibacterial spectrum of the *Listeria* phage LIS04^*a*^

Bacterial strain	Plaque formation^*b*^	Medium^*c*^
Gram-positive bacteria		
*Listeria innocua-*type strains		
ATCC 51742	+	BHI	
ATCC 33090	−	BHI	
KCTC 3586	*−*	BHI	
*Listeria monocytogenes-*type strains			
*L. monocytogenes* ATCC 19111	−	BHI	
*L. monocytogenes* ATCC 19115	−	BHI	
*L. monocytogenes* ATCC 15313	−	BHI	
*L. monocytogenes* DH-1	−	BHI	
*L. monocytogenes* KCCM 40307	−	BHI	
*L. monocytogenes* LM-1 12	−	BHI	
*L. monocytogenes* LM-1 16	−	BHI	
*L. monocytogenes* NCCP 15743	−	BHI	
*L. monocytogenes* 20800818	−	BHI	
*L. monocytogenes* 20800013	−	BHI	
*Bacillus* serotype strains			
*Bacillus cereus* ATCC 10876	−	LB	
*Bacillus cereus* KCCM 40133	−	LB	
*Bacillus subtilis* ATCC 11774	−	LB	
*Bacillus subtilis* KCCM 11315	−	LB	
*Bacillus subtilis* KCTC 2217	−	LB	
*Bacillus thuringiensis* KCTC 3452	−	LB	
Other strains			
*Enterococcus faecalis* ATCC 10100	−	LB	
*Staphylococcus aureus* ATCC 29213	−	TSB	
*Lactobacillus brevis* ATCC 14869	−	MRS	
*Lactobacillus plantarum* ATCC 8014	−	MRS	
*Lactobacillus rhamnosus* ATCC 53103	−	MRS	
Gram-negative bacteria			
*Cronobacter sakazakii* ATCC 29544	−	TSB/TSA	
*Escherichia coli* O157:H7 ATCC 35150	−	LB	
*Klebsiella pneumoniae* KCTC 2242	−	LB	
*Salmonella enterica* Typhimurium KCTC 1425	−	LB	
*Shigella flexneri* KCTC 2517	−	LB	

^*a*^ The spot test (5) as used to determine the host range against 20 bacterial strains. Phage lysate (1 × 10^8^ PFU) was spotted onto the bacterial lawns and incubated overnight at 37°C. The efficiency of plating (EOP) was measured as the ratio between the number of plaques on the test bacteria and the number of plaques of *L. innocua* ATCC 51742.

^*b*^ +, EOP of less than 1; −, no susceptibility to phage LIS04.

^*c*^ BHI, brain heart infusion; LB, lysogeny broth (also known as Luria–Bertani); MRS, De Man, Rogosa and Sharpe; TSA, tryptic soy agar; TSB, tryptic soy broth.

Phage DNA was extracted from a 10^10^ PFU/mL stock using the phenol-chloroform method and ethanol precipitation (5). Libraries were prepared with the TruSeq Nano DNA Kit (Illumina, USA) and sequenced using the Illumina MiSeq platform (300 bp paired-end reads). The sequencing run produced 1,787,366 reads with a total yield of 537,997,166 bp. Read quality was assessed using FastQC v.0.11.9 (https://github.com/s-andrews/FastQC). Adapter sequences and low-quality reads (*Q* < 20) were removed using Trimmomatic v.0.39 (7), resulting in 157,549 high-quality reads retained. The genome was assembled using the *de novo* assembler SPAdes v.3.15.2 (8). Gene annotation was performed using RAST (9), GeneMarkS (10), and FgenesV (Softberry Inc., http://linux1.softberry.com/berry.phtml), while functional open reading frames (ORFs) were analyzed via InterProScan (11) and BLASTp (12). Genome architecture was visualized using GeneScene v.0.99.8.0 (DNASTAR, USA). The lysogenic formation factors and antimicrobial-resistance genes of LIS04 were investigated using VFDB (BLASTn, *E* value <0.01) (13) and CARD-RGI v.6.0.3 (14), respectively. Comparative genomic analysis was performed using BLASTn (12).

**Fig 1:**
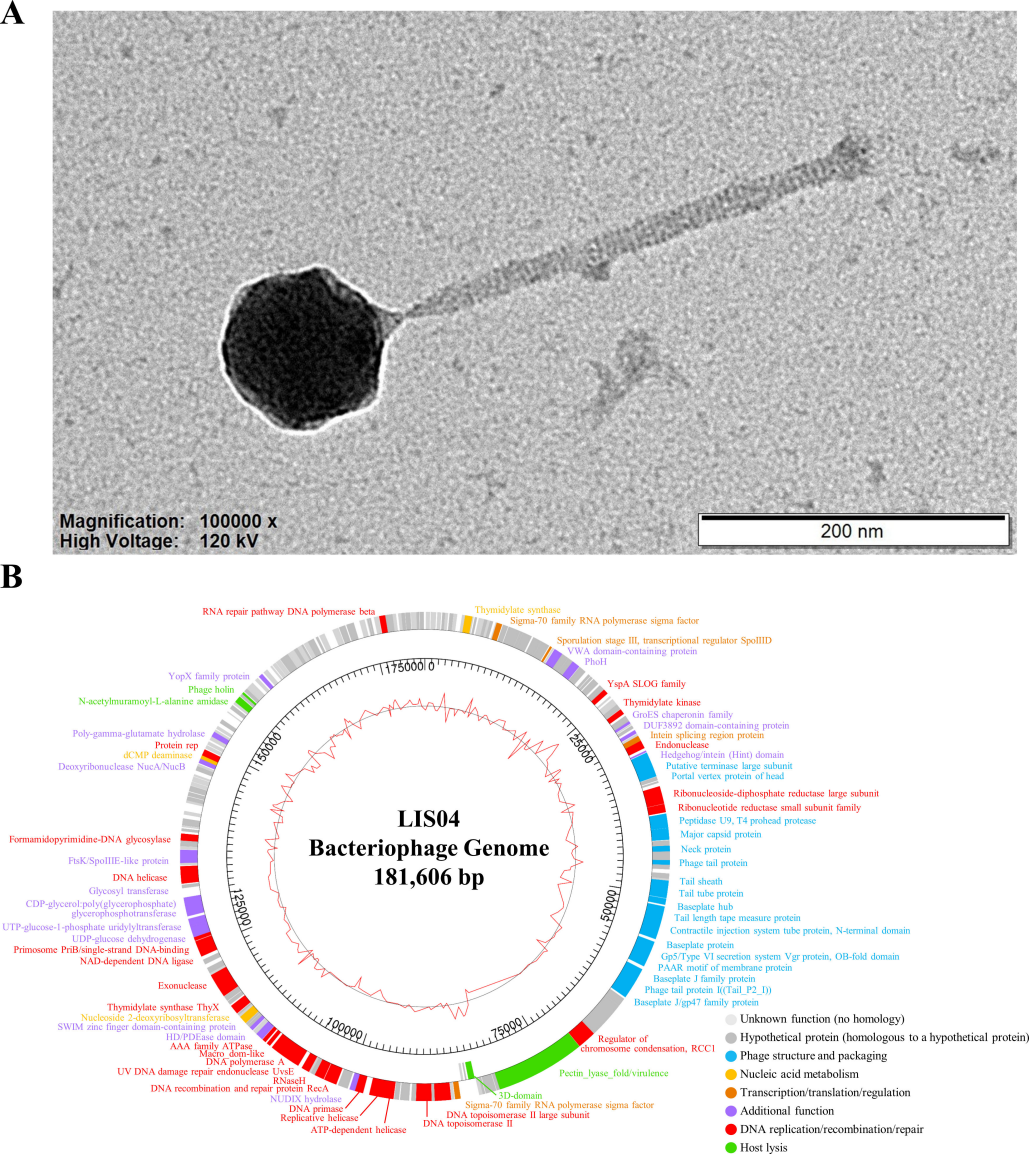
Morphological and genetical characterizations of the *Listeria* phage LIS04. (**A**) Transmission electron microscopy image of *Listeria* phage LIS04. The phage was negatively stained with 2% uranyl acetate (pH 4.5) and visualized under an energy-filtering transmission electron microscope (Libra 120 model; Carl Zeiss, Oberkochen, Germany) at an accelerating voltage of 120 kV. The scale bar indicates the magnification size of 200 nm. (**B**) The genome map of the complete genome of phage LIS04. The outer circle indicates the predicted open reading frames, and specific colors represent the functional category: phage structure and packaging (blue), nucleic acid metabolism (yellow), transcription/translation/regulation (orange), DNA replication/recombination/repair (red), host lysis (green), additional function (purple), unknown function (no homology, light gray), and hypothetical protein (homologous to a hypothetical protein, dark gray). The inner circle with a red line indicates the guanine-cytosine content. Scale units are base pairs.

In Fig. 1A, transmission electron microscopy analysis showed LIS04 has an isometric capsid (90.67 ± 3.16 nm, *n* = 15) and a long contractile tail (262.97 ± 4.58 nm, *n* = 15), classifying it within the *Caudoviricetes* class (15). LIS04 produced turbid plaques, with a diameter of approximately 7.47 ± 0.35 mm (*n* = 15). Host range testing revealed strict specificity for *L. innocua* ATCC 51742.

The LIS04 genome consists of 181,606 bp of double-stranded DNA (guanine-cytosine content: 37.09%) and encodes 217 ORFs, 70 (32.26%) of which were assigned putative functions in six categories (Fig. 1B). No genes associated with lysogeny, toxins, or antibiotic resistance factors were detected, supporting its lytic properties.

Comparative genomic analysis showed LIS04 shares the highest identity with *Bacillus* phage SP-15 (84.99%) and phiAFATE (79.34%), though with minimal query coverage (<2%), indicating significant genetic divergence. This suggests LIS04 represents a previously uncharacterized *Listeria*-infecting phage, though further phylogenetic studies are needed for precise taxonomic classification (3, 4).

In conclusion, LIS04 enhances our understanding of *L. innocua*-specific phages and shows potential for diagnostic and biocontrol applications, pending further functional validation.

## Data Availability

The complete genome sequence of Listeria phage LIS04 was deposited in GenBank under accession number OQ999172. The associated BioProject, BioSample, and Sequence Read Archive accession numbers are PRJNA1235970, SAMN47377773, and SRR32729517, respectively.
